# P05.37. Assessing patient perspectives on quality of care at community acupuncture clinics

**DOI:** 10.1186/1472-6882-12-S1-P397

**Published:** 2012-06-12

**Authors:** K Tippens, M Chao, E Connelly, A Locke

**Affiliations:** 1 National College of Natural Medicine, Portland, USA; 2 Osher Center for Integrative Medicine, San Francisco, USA; 3 Multnomah County Health Department, Portland, USA; 4 Outside In, Portland, USA

## Purpose

Community acupuncture is a recent innovation in acupuncture service delivery that provides low-cost treatments in group-based settings. Recent data suggests improved access to acupuncture through the community acupuncture model. However, there is a paucity of research on quality of care provided at community acupuncture clinics. The aim of this study was to assess patients’ perspectives on the quality of care received through community acupuncture clinics.

## Methods

A cross-sectional survey of 478 patients at two community acupuncture clinics in Portland, Oregon collected patient demographics, reasons for use, and satisfaction with treatment. In an open-ended survey question, respondents were invited to share additional comments about their experiences with community acupuncture. Qualitative analysis of open-ended survey responses was conducted using a grounded theory approach and Donabedian’s triad framework on quality of care (structure, process, outcomes).

## Results

Qualitative analysis of written comments identified two primary themes that elucidate patients’ perspectives on quality of care: (1) comparing community acupuncture to other healthcare delivery models, and (2) patients engaging in their own health care. Patients perceive the care they receive through community acupuncture as high quality compared to conventional medicine and to individual acupuncture treatment. Patients identify unique aspects of receiving quality care through community acupuncture including: structures that facilitate access; processes that make treatments more comfortable and effective; and holistic outcomes including physical improvements, enhanced quality of life, and empowerment.

## Conclusion

The group setting, community-based locations, and low cost of this model potentially reduce access barriers for those who might not otherwise consider acupuncture. These themes provide insight into patient values and experiences unique to community acupuncture, and are discussed here in the context of quality of care.

